# Case report: A novel third-generation anti-CD19/CD22 CAR T-cells combined with auto-HSCT for relapsed Burkitt lymphoma

**DOI:** 10.3389/fimmu.2024.1497736

**Published:** 2024-12-09

**Authors:** Xiaodan Luo, Ao Chen, Le Qin, Robert Weinkove, Rong Zhao, Ting Ye, Sihui Chen, Jianli Tang, Jianbo Liu, Jiayu Huang, Boyun Shi, Danyun Yuan, Huo Tan, Dajiang Qin, Zhaoyang Tang, Peng Li, Runhui Zheng

**Affiliations:** ^1^ The Fifth Affiliated Hospital, Guangzhou Medical University, Guangzhou, China; ^2^ China-New Zealand Joint Laboratory on Biomedicine and Health, Key Laboratory of Immune Response and Immunotherapy, Guangdong Provincial Key Laboratory of Stem Cell and Regenerative Medicine, GIBH-HKU Guangdong- Hong Kong Stem Cell and Regenerative Medicine Research Centre, GIBH-CUHK Joint Research Laboratory on Stem Cell and Regenerative Medicine, Institute of Drug Discovery, Guangzhou Institutes of Biomedicine and Health, Chinese Academy of Sciences, Guangzhou, China; ^3^ Cancer Immunotherapy Program, Malaghan Institute of Medical Research, Wellington, New Zealand; ^4^ Department of Automation, Tsinghua University, Beijing, China

**Keywords:** relapsed/refractory Burkitt lymphoma, CAR T-cell therapy, autologous hematopoietic stem cell transplantation, CD19/CD22 dual target, immunotherapy

## Abstract

This study explores a novel therapeutic strategy for relapsed/refractory (R/R) Burkitt lymphoma (BL) by integrating autologous hematopoietic stem cell transplantation (ASCT) with tandem anti-CD19/CD22 chimeric antigen receptor (CAR) T cell therapy. A 20-year-old Asian male with refractory BL, whose lymphoma had not responded to multiple chemoimmunotherapy regimens, received myeloablative ASCT followed three days later by infusion of a novel third-generation CAR T cells engineered with CD28 and CD3ζ signaling domains, along with a TLR2 costimulatory domain. This resulted in sustained complete remission at the 306-day follow-up, without experiencing any severe complications. This case suggests that combining myeloablative ASCT with tandem anti-CD19/CD22 CAR T cell therapy could be an effective approach for R/R BL, warranting further clinical validation.

## Introduction

Burkitt lymphoma (BL) is a highly aggressive B-cell non-Hodgkin lymphoma. Although responses to first-line therapy are high, relapsed or refractory (R/R) BL, carries a dismal prognosis with a median survival < 3 months, and fewer than 5% of patients surviving longer than two years (1). The few durable remissions reported for relapsed Burkitt lymphoma employ chemoimmunotherapy followed by autologous or allogeneic stem cell transplantation (2).

Chimeric antigen receptor (CAR) T-cell therapies directed against the B-cell antigen CD19 have been widely used as a salvage approach for R/R CD19^+^ acute lymphoblastic leukemia. Patients with BL were excluded from key lymphoma CAR T-cell registration trials, however, likely owing to the challenges that very rapid tumor growth presents to successful CAR T-cell manufacture and delivery. A lack of response to, or relapse following, CD19-directed CAR T-cell therapy for large cell lymphoma is common, due in part to downregulation of CD19 on tumor cells (3, 4). While subsequent treatment with CAR-T cells targeting the alternative B-cell antigen CD22 can result in clinical responses, these responses are often brief (5, 6). The disappointing results of single-targeting CAR-T cells in challenging disease settings have paved the way for the development of CAR-T cell therapies with specificity for two or more antigens (7–9). Liu, et al. sequentially infused CAR-T cells targeting CD19, CD22, and CD20 to 23 children with R/R BL, achieving a complete remission (CR) rate of 91% (10). However, this strategy requires manufacture of multiple CAR T-cell products. Another strategy is to combine multiple antigen specificities within a single CAR construct. We developed third-generation tandem anti-CD19/CD22 CAR T-cells employing CD28 and CD3 zeta (CD3ζ) intracellular signaling domains with a novel TLR2 costimulatory domain. Here, we report a case of successful treatment of an adult with R/R BL using myeloablative auto-HSCT followed by this new CAR T-cell therapy.

## Case presentation

**Figure 1:**
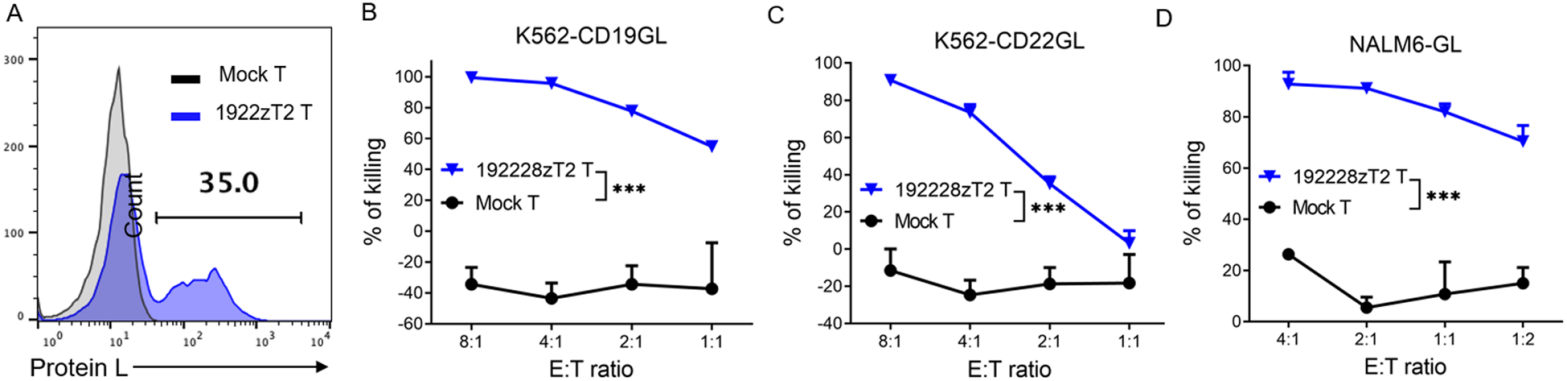
**(A)** The transduction efficiency of 1922zT2 T cells. **(B–D)**. The cytotoxicity activity of 1922zT2 T and mock T cells against CD19+ K562-CD19GL **(B)**, CD22^+^ K562-CD22GL **(C)** and CD19^+^CD22^+^ NALM6-GL **(D)** target cells *in vitro*. ALL target cells expressed GFP and Luciferase (GL). Data are presented as mean ± SD. *p* Values of B, C and D were calculated by two-way ANOVA with Tukey’s multiple comparisons test. ***p < 0.001.

We developed a novel tandem CAR, termed 192228zT2. This CAR incorporates humanized single-chain variable fragments (scFv) targeting both CD19 and CD22 extracellularly, and intracellularly incorporates a CD28 costimulatory domain, a Toll-like receptor 2 intracellular domain that is known to enhance the antitumor efficacy and migratory capacity of CAR-T cells (11–13), and a CD3ζmotif. The project was approved by the ethics committee of Guangzhou Medical University (GYWY-G2024-02), and the patient gave informed consent. The 192228zT2 cells were manufactured at the Good Manufacturing Practice (GMP) facility of Guangdong Zhaotai Cell Biology Technology Ltd. Briefly, T cells were isolated from peripheral blood mononuclear cells (PBMCs) using CliniMACS CD4 and CD8 reagents (Miltenyi Biotec). These cells were activated with MACS GMP T Cell Transact (Miltenyi Biotec). Subsequently, cells were transduced using a lentiviral vector encoding the 192228zT2 CAR, and the transduction efficiency was measured through protein L staining (**Figure 1A**). CAR T-cells were expanded in the presence of recombinant human IL-2 and harvested once the cell quantity met dosage requirements. *In vitro* killing assays demonstrated the efficient lysis of CD19-overexpressing K562 cells (K562-CD19GL) and CD22-overexpressing K562 cells (K562-CD22GL) by 192228zT2 T cells (**Figures 1B, C**), indicating the function of both anti-CD19 and anti-CD22 single-chain variable fragments (scFvs). Furthermore, 192228zT2 T cells exhibited effective killing of the human B-cell acute lymphoblastic leukemia (B-ALL) cell line, NALM6-GL, which expresses both CD19 and CD22 (**Figure 1D**).

**Figure 2:**
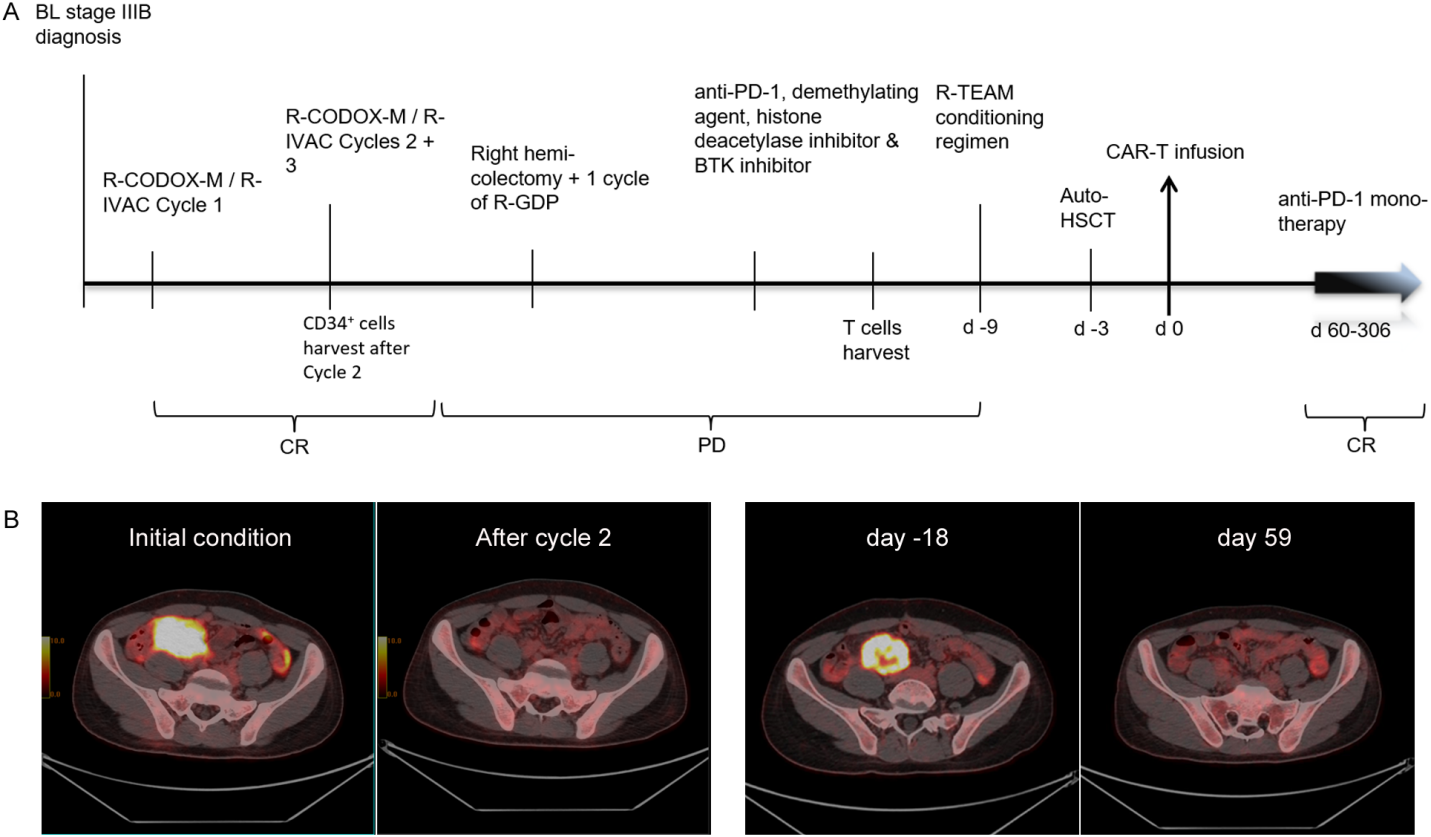
**(A)** Time-line of disease evolution and therapeutic interventions. **(B)**. PET/CT images at different time points: baseline, after 2 cycles of chemotherapy (CR), on day -18, and on day +59 following CAR T-cell therapy.

A 20-year-old Asian male had been diagnosed with stage 3B BL, presenting with abdominal and pelvis lymphadenopathy. The patient was HIV-negative at diagnosis, and the tumor tissue was positive for the t (8, 14) IGH/MYC translocation with a proliferation index (Ki67) of 95%. Tumor cells were negative for Epstein-Barr virus-encoded RNA (EBER) and next-generation sequencing (NGS) of lymphoma-related genes indicated a class I mutation of TP53 p.E258fs. A first cycle of R-CODOX-M/R-IVAC (R-CODOX-M: rituximab, cyclophosphamide, vincristine, doxorubicin and methotrexate; R-IVAC: rituximab, ifosfamide, etoposide and cytarabine), resulted in complete metabolic response on (18)F-FDG PET/CT (Deauville 5-point score 2). Following two more cycles, CD34^+^ cells (10.86 × 10^6^/kg) were harvested in preparation for a future auto-HSCT. However, a repeat PET/CT showed disease progression in the ileocecal mesenteric region. Right hemicolectomy was followed by one cycle of R-GDP (rituximab, gemcitabine, cisplatin and dexamethasone) and a cycle of a combination treatment comprising an anti-PD-1 antibody, demethylating agent, histone deacetylase inhibitor and Bruton tyrosine kinase (BTK) inhibitor, but disease continued to progress, with multiple new lesions in the mesentery and pelvic peritoneum. Re-biopsy of enlarged pericolic lymph nodes confirmed refractory BL and indicated partial CD19 expression (in 50% of tumor cells) and uniform expression of CD22 (in 100% of tumor cells). A decision was made to treat with 192228zT2 CAR T-cell therapy followed by auto-SCT. Time-line of disease evolution and therapeutic interventions were shown in **Figure 2A**. PET/CT images at different time points were shown in **Figure 2B**.

**Figure 3:**
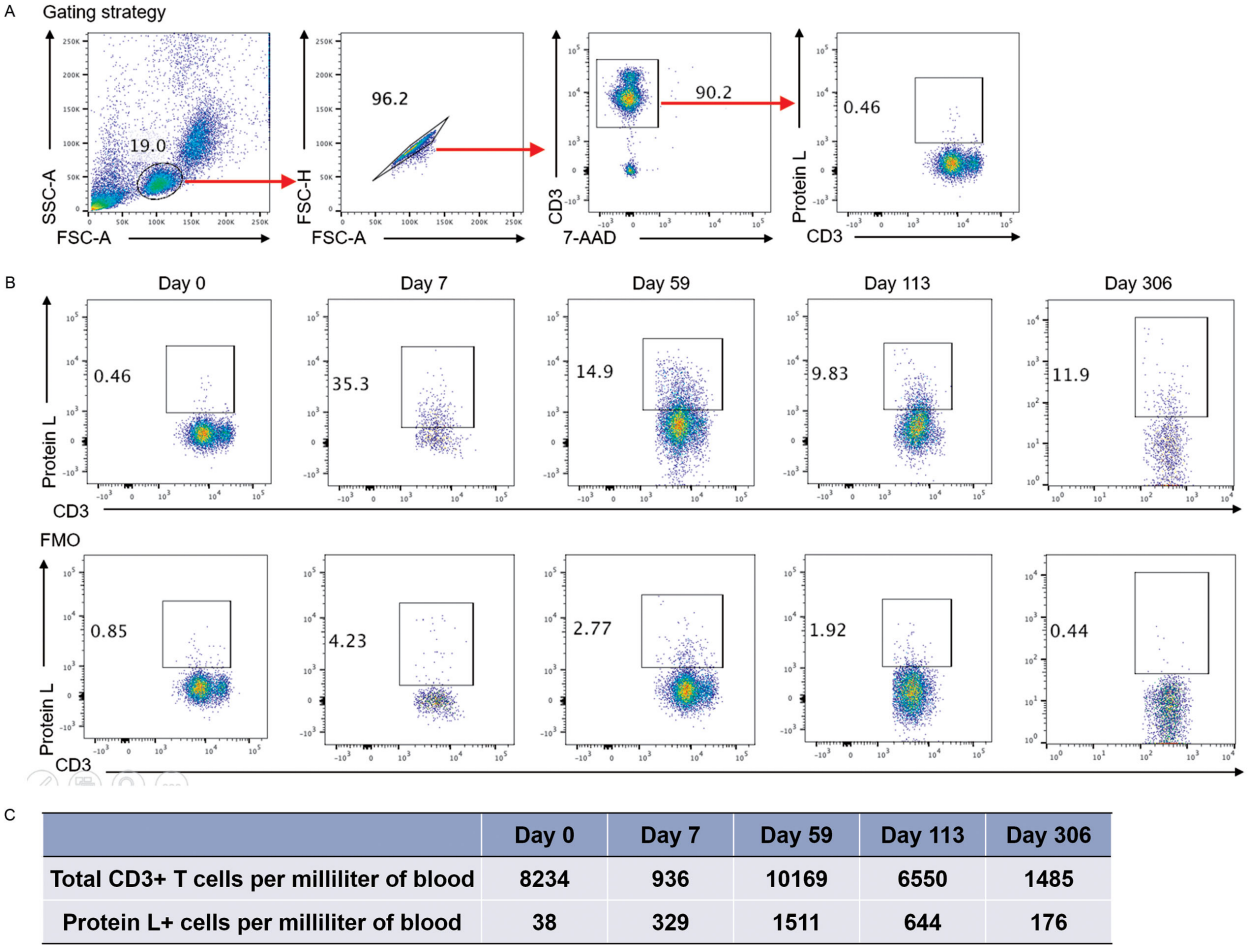
**(A)** Gating strategy for detecting CAR-T cells. The flow cytometry staining panel includes CD3-PE-Cy, 7-AAD, Protein L-Biotin, and either APC-streptavidin or PE-streptavidin. **(B)**. Flow Cytometry of Peripheral Blood: Blood samples were collected on day 7, 14, 28, 59, 113 and 306 days, and the presence of CAR T-cells was detected by protein L labeling within the CD3^+^ T-cell population. Fluorescence Minus One (FMO) control were applied at each time point to validate the threshold and ensure accurate detection. **(C)**. Total CD3^+^ cells and Protein L+ cells per milliliter of blood at all time points.

After successful 192228zT2 T manufacture, the patient received a myeloablative R-TEAM (rituximab, thiotepa, etoposide, cytarabine and melphalan) conditioning regimen, which replaced the lymphodepletion (LD) regimen, followed by auto-HSCT. Three days after auto-HSCT, 1.2 × 10^6^ CAR T cells per kilogram 192228zT2 CAR T-cells were administered. Grade 1 cytokine release syndrome (CRS) was diagnosed, and tocilizumab (8 mg/kg, administered every day to every 8 hours) was delivered for persistent fevers, which resolved by day 7. The patient achieved neutrophil engraftment on day 19 and platelet engraftment on day 22, No infectious complications were observed. PET/CT at day 59 revealed complete metabolic remission (Deauville 5-point score 3) (**Figure 2B**). Following this, the patient continued maintenance monotherapy with Tislelizumab (anti-PD-1 monoclonal antibody, BeiGene) every 28 days. We detected the proportion of 192228zT2 T in peripheral blood after injection. Gating strategy for detecting CAR-T cells is shown in **Figure 3A**. The result showed that 192228zT cell began to expand on day 7 (3.66%) and persist for an extended period (**Figure 3B**). Total CD3^+^ cells and Protein L+ cells per milliliter of blood at all time points (**Figure 3C**). As at day 306, the patient is in ongoing complete remission.

## Discussion

BL disease progression after multi-line chemotherapy is a catastrophic event lacking a defined effective treatment approach. The expected therapeutic efficacy of HSCT is not optimistic, since on one hand, disease progression indicates high chemo-resistance, which diminishes the potential benefits from heavy conditioning regimen before HSCT. On the other hand, HSCT showed poor outcome in BL patients, and it is unclear whether there is a strong graft-versus-lymphoma effect following allo-SCT (14, 15). The Center for International Bone and Marrow Transplantation Research (CIBMTR) reported the outcomes of 241 patients undergoing HSCT for BL, and those with R/R disease had a 5-year PFS and OS of 27% and 31% for auto HSCT, and only 19% and 20% for allogenic HSCT, respectively (14).

While CAR T-cell therapy is a promising modality of treatment, patients with R/R BL are liable to experience disease progression during CAR T-cell manufacture, LD chemotherapy and/or before CAR T-cells can proliferate and exert effector activity *in vivo.* There’s an urgent need for new treatment strategies. Here, we describe a novel approach that combines three strategies: (1) the use of myeloablative chemotherapy followed by auto-HSCT to both debulk the BL and provide lymphodepletion; (2) the administration of CAR T-cells shortly after auto-HSCT, before engraftment; (3) use of a new third-generation tandem CD19/CD22 CAR T-cell product to maximize antitumor efficacy, and (4) maintenance anti-PD1 therapy in an effort to prevent CAR T-cell exhaustion.

CAR-T cell treatment failure have been reported for lymphomas compared with ALL patients. The mechanism may include rapid lymphoma progression, antigen escape, immunosuppressive tumor microenvironment (TME) and CAR-T cell exhaustion (3, 16–18). By combining a myeloablative auto-HSCT with CAR T-cell therapy, the opportunities for rapid lymphoma progression were diminished. Noting the partial CD19 expression on the BL cells at the time of lymphoma progression, 1922zT2 T cells used in this study, incorporating humanized scFvs targeting both CD19 and CD22, may have reduced the risk of antigen escape. Incorporation of a TLR2-derived intracellular domain has the potential to promote specific activation and expansion of T cells, showing improved TME and antitumor efficacy of CARs (11, 12, 19). T-cell exhaustion and an immunosuppressive TME may contribute to CAR T-cell failure, and PD-1 blockade has been reported to improve the antitumor activity of CAR-T cells (20–22). In the phase 1b PORTIA study (NCT03630159), the ORR of patients with R/R DLBCL treated with tisagenlecleucel in combination with pembrolizumab was 50% and the CR rate was 33.3% (23). A phase 1/2a trial (NCT02650999), evaluating pembrolizumab for B-cell lymphomas relapsing after or refractory to CD19-directed CAR T-cell therapy, showed a best ORR of 25%. In this trial, CAR T-cell profiling before and after pembrolizumab treatment was analyzed and high levels of inhibitory receptors such as LAG-3, Tim-3 and CTLA-4 before permbrolizumab were found to be decreased after PD-1 blockade (21). Therefore, the pre-emptive use of anti-PD1 therapy in this case may have improve the CAR T-cell activity.

With auto-HSCT alone, it is difficult for patients to achieve CR, while with CAR T-cell therapy alone, a high tumor burden increases the risk of severe cytokine release syndrome (CRS) and affects hematopoiesis. Combining myeloablative auto-HSCT with CAR T-cell therapy could effectively reduce tumor burden, diminish the immunosuppressive microenvironment, and therefore enhance CAR T-cell function, promote engraftment, and support immune reconstitution (19, 24, 25). Despite the potential risks of CRS or Immune Effector Cell-Associated Neurotoxicity Syndrome (ICANS) caused by an inflammatory environment, we did not observe severe CRS or ICANS, nor was hematopoietic reconstitution delayed in this case.

In conclusion, the combination of auto-HSCT followed by 192228zT2 therapy offers a promising new approach for treating R/R BL.

## Data Availability

The raw data supporting the conclusions of this article will be made available by the authors, without undue reservation.
